# Automatic classification and neurotransmitter prediction of synapses in electron microscopy

**DOI:** 10.1017/S2633903X2200006X

**Published:** 2022-07-29

**Authors:** Angela Zhang, S. Shailja, Cezar Borba, Yishen Miao, Michael Goebel, Raphael Ruschel, Kerrianne Ryan, William Smith, B. S. Manjunath

**Affiliations:** 1 Vision Research Laboratory, University of California, Santa Barbara, Santa Barbara, California, USA; 2 Neuroscience Research Institute, University of California, Santa Barbara, Santa Barbara, California, USA; 3 Department of Molecular, Cellular, and Developmental Biology, University of California, Santa Barbara, Santa Barbara, California, USA; 4 Meinertzhagen Laboratory of Invertebrate Neurobiology, Dalhousie University, Halifax, Nova Scotia, Canada.

**Keywords:** *Ciona*, computer vision, electron microscopy

## Abstract

This paper presents a deep-learning-based workflow to detect synapses and predict their neurotransmitter type in the primitive chordate *Ciona intestinalis* (*Ciona*) electron microscopic (EM) images. Identifying synapses from EM images to build a full map of connections between neurons is a labor-intensive process and requires significant domain expertise. Automation of synapse classification would hasten the generation and analysis of connectomes. Furthermore, inferences concerning neuron type and function from synapse features are in many cases difficult to make. Finding the connection between synapse structure and function is an important step in fully understanding a connectome. Class Activation Maps derived from the convolutional neural network provide insights on important features of synapses based on cell type and function. The main contribution of this work is in the differentiation of synapses by neurotransmitter type through the structural information in their EM images. This enables the prediction of neurotransmitter types for neurons in *Ciona*, which were previously unknown. The prediction model with code is available on GitHub.

## Impact Statement

The working of a brain is an emergent property of the attributes and connectivity of its constitutive parts—the neurons. The field of connectomics seeks to describe these features on a nervous systemwide scale, because only then can the researcher conceptualize, model, and manipulate complete neural circuits driving behavior. Experimentally describing the enormous number of synaptic connections within a nervous system in detail is a daunting task, and is limited by the current technology to animals with small nervous systems. One such animal is the primitive Chordate *Ciona.* The *Ciona* connectome, which was derived by serial-section electron microscopy (EM), describes the 177 neurons, and 6,618 synapses, that make up the central nervous system of a *Ciona* larva. While the resulting EM-derived connectivity matrix isely essential starting point for circuit analysis, it does not readily reveal essential properties of the neurons, such as excitatory, inhibitory, or modulatory synaptic connections. The goal of the current work is to determine if neurotransmitter types can be distinguished through structural information in synaptic EM images. This enables the prediction of neurotransmitter types for neurons in *Ciona*, which were previously unknown, and paves a way for streamlined annotation of EM images of synapses.

## 1. Introduction

**Figure 1.**
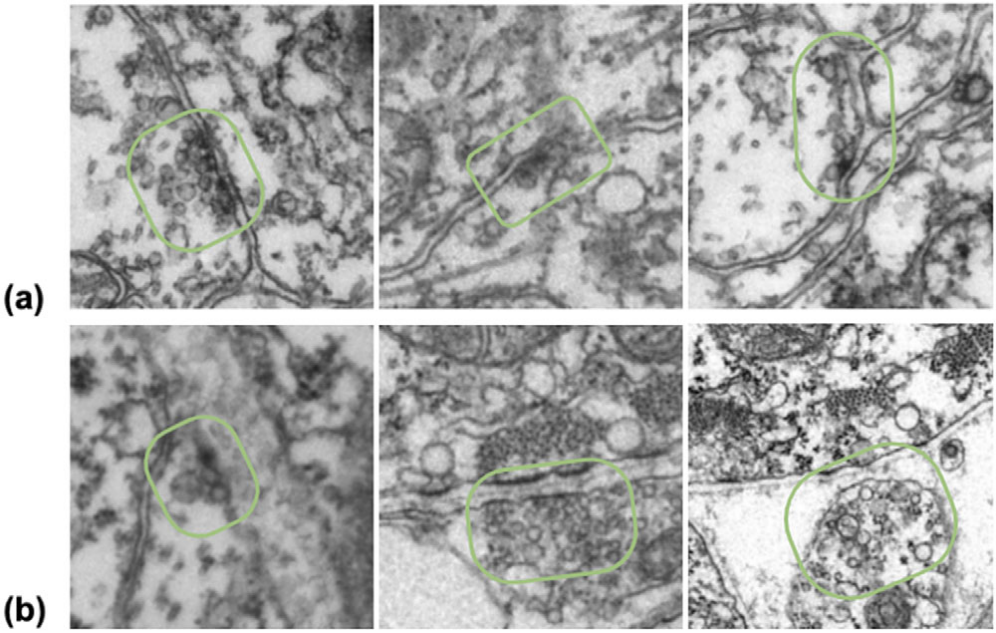
Examples of (a) inhibitory and (b) excitatory synapses. The synaptic region is circled in green. It can be seen that there are varying vesicle counts and sizes, as well as little visible postsynaptic density.

We propose a deep-learning, convolutional neural network to detect *Ciona intestinalis* (*Ciona*) synapses via image patch classification from serial-section-transmission electron microscopic (EM) images, as well as a multimodal method of predicting neurotransmitter type based on several modalities of data obtained in different ways—EM imaging, light microscopy of in situ hybridization, and behavioral observation experiments. *Ciona* is of interest to neuroscientists because of its close evolutionary relationship to vertebrates and small nervous system. Moreover, it is one of the very few animals for which a complete synaptic connectome is available^(^^1^^)^. An essential aspect of constructing a connectome, a comprehensive map of neurons and their connections in the brain, is to identify the synapses that form chemical and electrical communication links between neurons^(^^2^^)^. Traditionally, the classification of synapses is completed manually through expert analysis of thousands of images. This is a time-consuming process that can take thousands of hours for one specimen. For example, the first *Ciona* connectome was constructed manually over a period of 5 years from serial-section EM images^(^^1^^)^. Another crucial but missing component of understanding the connectome is the classification of the function and properties of each synapse. One of the most important properties is neurotransmitter use. While some experimental methods, such as in situ hybridization, can identify cells, or clusters of cells, expressing transcripts indicating neurotransmitter use^(^^3^^)^, the resolving power of light microscopy cannot reveal the connectivity of these neurons. Additionally, in cases with intermingled neurotransmitter types and high variability in neuron location across specimens, it may not be possible to find correspondence between neuron properties derived by in situ hybridization and neurons identified by serial EM in the course of constructing the connectome. Visual inspection of the synapses in *Ciona* EM images does not reveal any distinguishable differences between known excitatory and inhibitory neurons. Some examples are included in Figure 1. Like all chemical synapses, those in *Ciona* include the vesicle cluster in the presynaptic neuron, which varies in count and size, and the postsynaptic density, which also varies in size and density. No apparent pattern in these features is visible upon manual inspection.

In this paper, We define “Synapse Classification” to mean the classification of whether an image patch contains a synapse or not. We define “Neurotransmitter-Type Prediction” to mean the prediction of neurotransmitter type in an image patch already assumed to contain a synapse. We use manually annotated EM images of a *Ciona* larva to validate our findings. We also propose a deep-learning method to predict the neurotransmitter type of a neuron based on the appearance of its synapses in EM images, and generate Class Activation Maps^(4)^ from the prediction model to visualize the features that are important in synapse neurotransmitter-type prediction.

### 1.1. Contributions

Our main contributions are as follows.

1. An automated method to detect and localize synapses from EM data with high accuracy.
2. A method to predict the neurotransmitter class of neurons in *Ciona* based on its synapse structure. Creation and analysis of class activation maps based on grad-CAM^(^^4^^)^ from the neural network to derive the synaptic features that are identified as important to neurotransmitter-type prediction.
3. Model-based predictions of neurotransmitter class by cell, which are previously unknown, and can be used in further experiments to help determine the true neurotransmitter expression of said cells.

The methods and data are available upon request.

## 2. Prior Work

Previous work have reported successful application of computer vision methods for automatically detecting synapses in EM images of *Drosophila*, mouse, and rabbit neurons^(^^5^^–^^9^^)^. However, the synapses of these organisms contain unique features, which the systems rely on heavily. The algorithm for *Drosophila* synapse detection^(5,8,9)^ involves the distinctive t-shaped feature and postsynaptic density to detect the synapse, often with a 3D U-Net-inspired network^(^^10^^)^, while the algorithms for mouse synapse detection^(6,7)^ were created for cryogenic EM. One approach applies pixel-level classification and graph cut segmentation on EM images to identify potential synapses, then filters the potential synapses with a random forest classifier^(^^6^^)^. The classifiers are trained on manually annotated samples. A second approach finds handcrafted synaptic cleft features for the presynaptic and postsynaptic region, and uses LogitBoost to perform the final synapse detection^(^^7^^)^. A third publication describes the uses of a fusion of ribbon, cleft, and vesicle features of a rabbit retina synapse to detect retinal synapses through kernel learning^(^^11^^)^. Ribbons are not ubiquitous among synapses, and clefts are not always visible for different types of synapses, so this method would not work on all types of synapses.

Studies have qualitatively shown differences between excitatory and inhibitory synapses—namely that the vesicle shape and postsynaptic density appearance varied between the two functions^(^^12^^–^^14^^)^. However, these papers are mostly qualitative and do not provide predictive functionality. Furthermore, these studies do not analyze a large number of synapses, and are not directly translatable to *Ciona* synapses due to differences between species. More recently, synapse neurotransmitter-type prediction using a deep network was done on EM images of *Drosophila* neurons^(^^15^^)^. This body of work is most similar to the one presented in this paper, but the appearance of *Drosophila* synapses differs drastically from those of *Ciona.* Our previous study^(^^3^^)^ combined in situ hybridization with the existing connectome derived from EM to determine the neurotransmitter expression of neuron types, such as the photoreceptors. Point cloud matching was done to match relative cell locations in three dimensions between fluorescent microscopic and EM results. While we are successful in determining the neurotransmitter expression of individual cells belonging to the photoreceptors, we are unable to determine with certainty the neurotransmitter expression of individual cells belonging to the relay neurons (RNs) and other types. The present study aims to take steps toward resolving the neurotransmitter assignments in these ambiguous regions while applying neural network approaches to this problem.

## 3. Data

**Figure 2.**
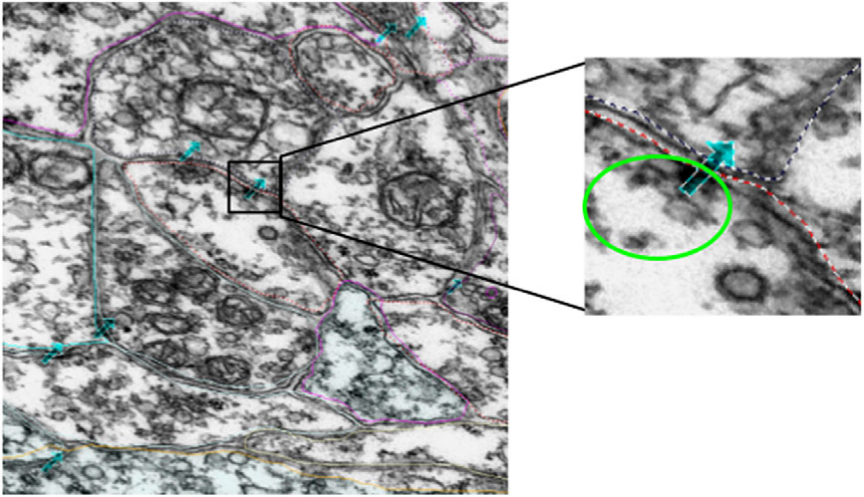
Example of manual annotations of synapses, indicated by the cyan arrows. The zoomed-in image shows a patch containing a synapse, with the vesicles encircled with bright green and the cell boundaries marked with dotted lines. The direction of the arrow does not indicate synaptic direction.

The data consisted of EM images from a total of 3,375 60-nm serial sections from the anterior brain vesicle to the motor ganglion of a *Ciona* larva. The sections were collected and imaged at 3.85-nm per pixel^(^^1^^)^. The data collected surpassed 1 TB. The original *Ciona* EM serial-section image dataset was collected, processed, and annotated using the program RECONSTRUCT^(^^16^^)^. The annotations we focused on are the synapse annotations (Figure 2), which are stored as points in three dimensions with a naming system to indicate the pre- and postsynaptic neurons. In order to facilitate analysis, python scripts were written to interface with the program and stored data and extract image patches corresponding to annotated regions. A series of geometric transformwereas used to co-register the annotation coordinates with the aligned 3D image stack coordinates. The RECONSTRUCT images used in this project are available upon request. The image processing and extraction steps are described in Section 4.

### 3.1. Data curation and annotation

**Figure 3.**
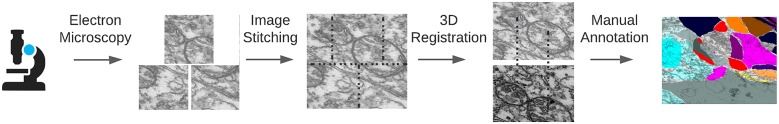
Illustration of data preparation method.

The EM images were scaled and aligned in the *z*-dimension while annotating the cellular components. Image scaling is specified by a pixel size (magnification) parameter, while alignment is represented by a nonlinear transformation associated with the image. Each transformation maps trace points or image pixels onto the section using a combination of basic functions representing an elementary motion such as translation, orientation, scaling, and deformation. We extract the underlying annotations (actual section coordinates in microns) from each section by combining these movement components in different proportions. Each image is associated with an independent transformation which determines the size and location of the element on the section. Applying the inverse transform on the contour point (*x*, *y*), we obtain the points (*x*’, *y*’), on which applying the forward image transformation brings the points to the original image domain. For our study, a synapse is represented by seven points forming an arrow, as shown in Figure 3. After getting the coordinates of these points on the original image domain, we determine the centroid of these points and extract a 500 × 500-dimensional patch around this centroid point. We perform this operation for all the synapse annotations to obtain approximately 25,000 total patches. The code to extract synapse points from EM data can be found at https://github.com/s-shailja/Ciona_EM.

The annotations we are working with indicate a general region with a 500 × 500 patch size within which one or more synapses are present, but the exact synapse boundaries were not part of the annotation in the dataset. The method for synapse localization would work for full-size images by taking overlapping patches, with localization done on the level of the image patch size.

## 4. Methods

**Figure 4.**
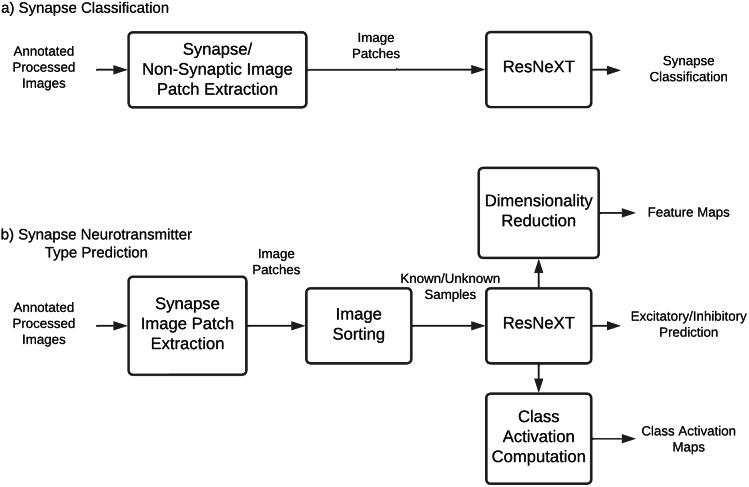
EM image processing flowcharts.

The major steps involved in *Ciona* synapse classification are shown in Figure 4. The synapse classification workflow includes image patch extraction and training of a ResNeXT network. The neurotransmitter prediction workflow is similar but sorts the images based on the assigned IDs^(^^1^^)^ of the presynaptic cell before training the deep learning network. Post-prediction class activation maps of the convolutional neural network are computed for ba etter understanding of important imaging features for neurotransmitter-type prediction. Feature maps are reduced to two-dimensional space and plotted to visualize the feature-space distance between synapses of various neurotransmitter types.

For synapse classification, the same training process is used as follows. A ResNeXt-50 network architecture^(^^17^^)^ pretrained on the ImageNet^(^^2^^)^ dataset was retrained on the extracted image patches. First, the last fully connected layer was replaced with a randomly initialized fully connected layer with an input of 2,048 and an output of 2. The output has two possible classes, with 0 being inhibitory and 1 being excitatory. The training was done in two sessions. First, all layers of the ResNeXt are “frozen,” with no gradient, except for the last fully connected layer. The model is trained for 100 epochs, and the best model is used for the second round of training. On the second round of training, the entire network is “unfrozen,” and every layer of the network is tuned with retraining for 200 epochs. The architecture of the deep network is shown in Table 1.

**Table 1.** ResNeXt-50 architecture.

Stage	Layers		Output
Conv1	7 × 7, 64	Stride 2	112 × 112
Conv2 (×3)	3 × 3 max pool	Stride 2	56 × 56
	1 × 1, 128	*C* = 32	
	3 × 3, 128		
	1 × 1, 256		
Conv3 (×4)	1 × 1, 256	*C* = 32	28 × 28
	3 × 3, 256		
	1 × 1, 512		
Conv4 (×6)	1 × 1, 512	*C* = 32	14 × 14
	3 × 3, 512		
	1 × 1, 1,024		
Conv5 (×3)	1 × 1, 1,024	*C* = 32	7 × 7
	3 × 3, 1,024		
	1 × 1, 2,048		
	Global average pool		
	1,000-d fc, softmax		Prediction (0 or 1)

### 4.1. Synapse classification

Image patches containing annotated synapses and image patches containing nonsynaptic structures were used for the training (80%) and testing (20%) sets. A total of 1,400 500 × 500 image patches containing synapses, and 1,392 500 × 500 image patches containing nonsynaptic structures, were used for training a synapse classification network. Nonsynaptic structures used were annotated variously as botrysomes, coated vesicles, basal bodies, and autophagosomes. Annotated structures that were avoided due to the possibility of synapses being in the same patch included terminals, vesicles, dense core vesicles, and gap junctions. Misclassified patches are analyzed visually for confounding factors.

### 4.2. Synapse neurotransmitter prediction

Image patches were grouped by presynaptic and postsynaptic cell ID^(^^1^^)^ before splitting into training, validation, and testing sets. A similarity computation was applied to each patch to ensure that no duplicates or extremely overlapping patches were used. Due to the limited number of synapses and known neuron groups available, we combined the two inhibitory neurotransmitter types (glycine and gamma-aminobutyric acid [GABA]) and the two excitatory neurotransmitter types (glutamate and acetylcholine). A total of 470 excitatory synapses and 338 inhibitory synapses were used for training a synapse classification network. A total of 1,246 excitatory synapses and 396 inhibitory synapses were used for testing. Each synapse was composed of 3–10 image patches in the *z*-dimension.

Nine neuron groups with four known neurotransmitter types, as determined by in situ hybridization^(^^3^^)^, were used in training and testing. Each presynaptic neuron had 1–41 associated synapses. Neurotransmitter class was predicted using the trained network on an additional 13 neuron groups with previously unknown neurotransmitter types. For each presynaptic neuron, a majority vote was made from the predictions of each synapse belonging to the neuron. Based on the strength of consensus, network confidence, and prior knowledge from in situ hybridization experiments^(^^3^^)^, predictions were made on the neurotransmitter type of neurons that surpassed a confidence interval described as follows. For each neuron, we tallied the number of predictions for each neurotransmitter valence. This tally is referred to as a vote. The valence with the most votes is chosen as the raw prediction of that presynaptic neuron. If the votes for each valence are close (e.g., inhibitory vote is not more than 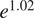 times of the excitatory votes) or that the total number of votes is less than 3, we determine the prediction to be inconclusive.

### 4.3. Class activation maps

The patterns picked up by the deep-learning network might provide insights to the features visible in the EM image that may connect synapse form to function.

A total of 512 × 512 image patches with labels are passed through the neurotransmitter prediction neural network. The final layer of our network uses global average pooling to go from a size of 16 × 16 × 2,048 to 2,048 features, then compute probabilities for each of the *N* classes using a fully connected layer without bias. For each pixel in the 16 × 16 feature map, we then compute the amount that this feature contributes to the output class. We apply the fully connected weights to the features at each pixel, cutting out the average pooling step. The basic idea for deriving class activation maps is described in Reference (18). After extracting the activation maps from the model, we use connected component analysis to find seed points, then employ a watershed algorithm to segment the activation map into disjoint regions to further analyze spatial and intensity information about the activations. Next, we removed regions that were smaller than a set threshold, which was set empirically to 5,000 pixels. Following this step, for each remaining connected region, we then calculated the *x*- and *y*-coordinates of the centroid for the region and the average activation intensity inside the region of interest.

### 4.4. Feature maps

To see how the image features detected by the deep network relate to neurotransmitter type, we perform t-distributed Stochastic Neighbor Embedding^(^^19^^)^ after principal component analysis (PCA) to visualize the features.

The t-SNE plot shows the feature projection of each individual synapse, not grouping the synapses by cell. The high-dimensional features from the output layer of the prediction network were reduced to 20 dimensions using PCA. The 20-dimensional feature is then further reduced to two dimensions using t-SNE, which better visualizes the clustering characteristics of the features. The features are grouped in various ways to gain more insight into how they are clustered.

## 5. Results

For synapse classification, a training accuracy of 0.99 and a testing accuracy of 0.98 were achieved. For training, 2,780 samples were used, with half of the samples containing synapses and half without synapses. For testing, 692 samples were used, also with a 50/50 split of synapse versus nonsynapse sample. There were no false negatives, but there were 13 false positives, which were detected by the model. Examples of some false positives are shown in Figure 12.

Performance of the network on neurotransmitter-type prediction is shown in Table 2 and Table 3. Precision is the number of true positives (TPs) divided by the sum of TPs and true negatives, and recall is TPs divided by the total number of positive samples. Precision determines how often selected items are relevant, and recall determines how often relevant items are selected.

**Table 2.** Automated neurotransmitter prediction performance—per synapse (train and test) and per cell.

	Precision (per synapse)	Recall (per synapse)	Precision (per cell)	Recall (per cell)
Inhibitory	0.98 train/0.98 test	0.93 train/0.91 test	1.0	0.95
Excitatory	0.96 train/0.93 test	0.99 train/0.99 test	0.98	1.0

**Table 3.** Automated synapse neurotransmitter-type performance breakdown by neurotransmitter type. As seen in the table, the performance for glycine was the worst, most likely due to the low number of cells and synapses available to train the model. Performance for GABA, acetylcholine, and glutamate were similar to each other. Each cell contains multiple 500 × 500 pixel patches of synapses spread throughout 3D space and may or may not be overlapping.

	Number of cells	Number of synapses	Accuracy (per cell)	Accuracy (per synapse)
Gly	3	22	0.67	0.71 train/0.6 test
GABA	19	457	1.0	0.92 train/0.95 test
Ach	19	292	1.0	0.97 train/1.0 test
Glut	23	342	1.0	1.0 train/0.98 test
Overall	64	1,113	0.98	0.95 train/0.97 test

*Abbreviation*: GABA, gamma-aminobutyric acid.

Of the failed predictions (all false positives), 10 cases were image patches annotated as coated vesicles, 2 cases were annotated as botrysomes, and 1 case was annotated as an autophagosome. Some representative patches are included in Figure 12. From the failed cases, it can be seen that they tend to contain cell boundaries and vesicles, which are features associated with synapses. The lack of false negatives is reassuring, as the goal of the classification network is to detect likely synapses for screening by experts, so false positives are better tolerated than false negatives.

**Figure 5.**
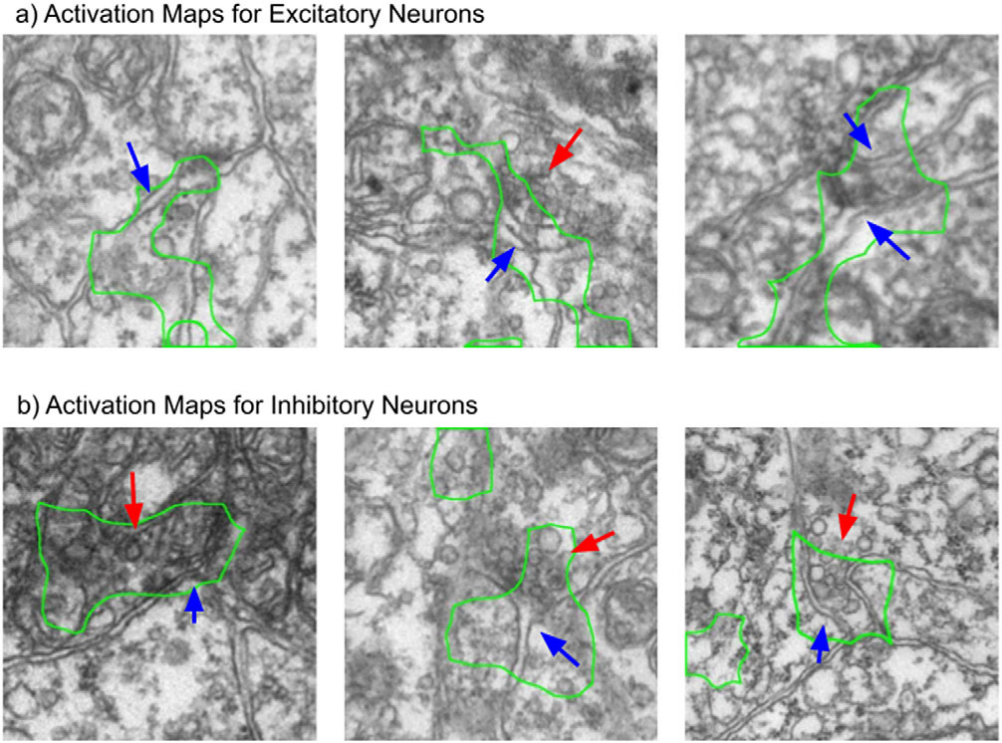
Processed class activation maps for excitatory and inhibitory synapse image patches. The network seems to pay more attention to the vesicles when predicting inhibitory neurons, and the cell boundary when predicting excitatory neurons. The blue arrows indicate cell boundaries, whereas the red arrows indicate vesicles. The green outline shows the main region of interest of the network.

To get a better idea of the features that are important to the neurotransmitter predictor network, we visualized the activation maps of the network on different classes, as described in Section 4. The results of the visualization show that cell boundaries, vesicles, and postsynaptic density are the main focus of the majority of the attention for the trained network. Some examples of activation maps are shown in Figure 5.

**Figure 6.**
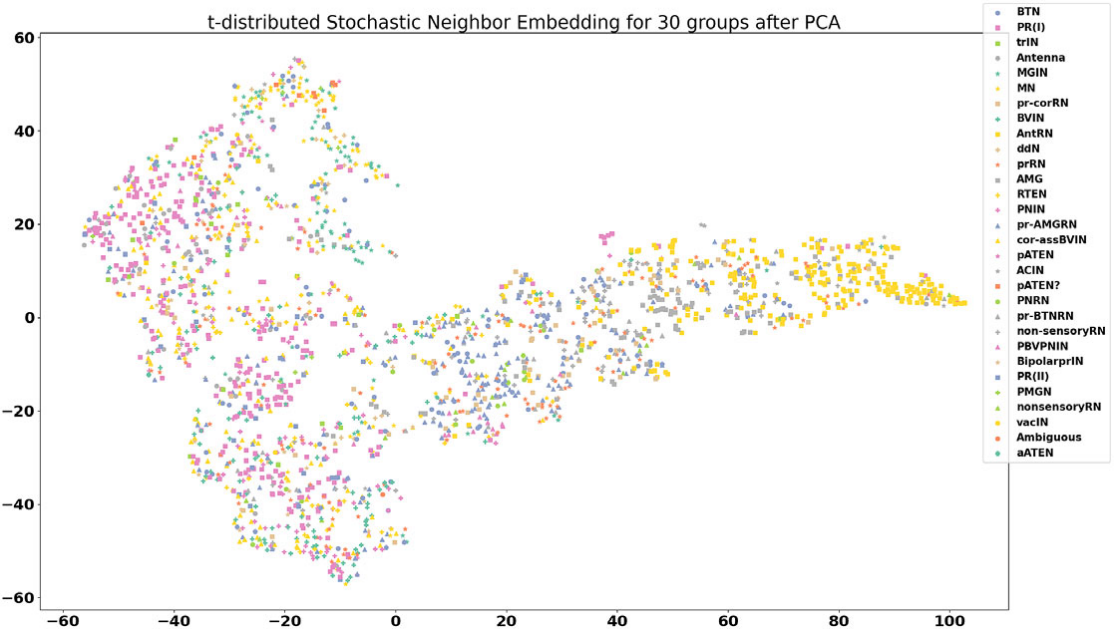
Visualization of features for 30 neuron groups after reduction to two dimensions using principal component analysis and t-distributed stochastic neighbor embedding. Each data point on the plot is the computed feature of a synapse, with synapses that span multiple frames averaged across all frames. There is quite a bit of intermingling of the features between cell groups, which is an encouraging sign that the model is picking up on differences not unique to each cell type.

**Figure 7.**
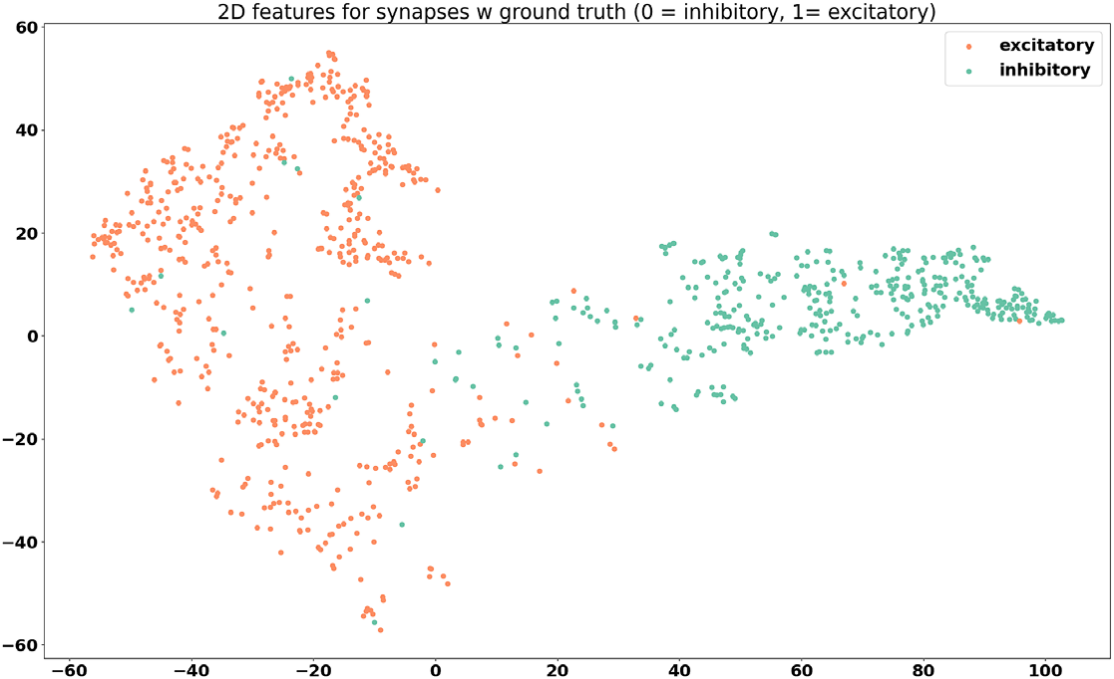
Feature visualization for synapses belonging to cells with known neurotransmitter type, grouped by valence. Two distinct groups can be seen.

**Figure 8.**
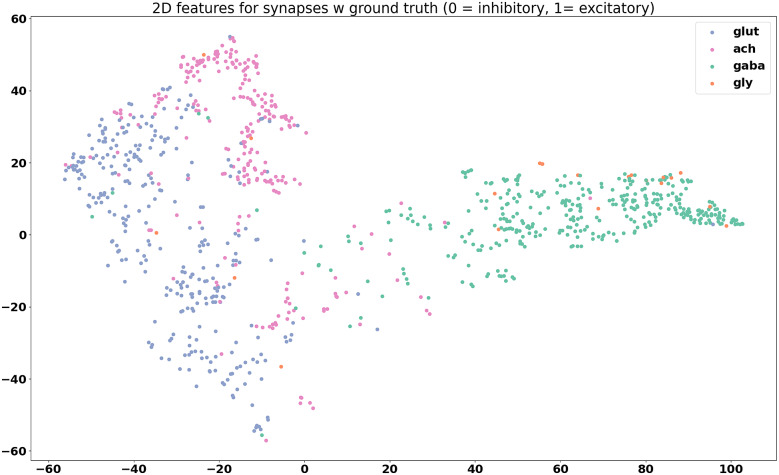
Feature visualization for synapses with known neurotransmitter type, grouped by neurotransmitter. Even though the prediction model was trained only to differentiate between excitatory and inhibitory synapses, it seems that the features tend toward separation by neurotransmitter type, with the exception of glycine, again likely due to the lack of training samples.

The feature maps derived from the model outputs are shown in Figures 6– 8.

From the feature maps, it is evident that the synapses are placed in clusters that mostly correspond to their neurotransmitter class, inhibitory, or excitatory. From Figure 8, it appears that the differences between different excitatory neurotransmitters (acetylcholine and glutamate) are also captured by the model, even though this information was not explicitly included during the training. From Figure 6, it can be seen that the features of synapses tend to be spread out throughout the feature space, and co-mingle among cell groups. This is promising, because this indicates that the model is picking up on differences between synapses that are more indicative of neurotransmitter class, rather than group-specific differences.

Table 5 shows the performance of the model on synapses belonging to cells of known neurotransmitter type. Some of these synapses were used for training, and others were used for testing. We tallied the number of predictions by presynaptic neuron type and valence (inhibitory and excitatory) in the training set. The tally is repeated for the testing set. The tally for each valence is referred to as a vote. The valence with the most votes is chosen as the raw prediction of that presynaptic neuron. Those are the values in the Predicted Valence section. We compared the valence of the raw prediction with the neurotransmitter type determined by in situ hybridization to calculate precision and recall.

Table 4 shows the predictions of the model on RNs of unknown neurotransmitter type. We noticed that the prediction model tends toward predicting more excitatory synapses in the RN group, since the observed average number of excitatory RNs from in situ hybridization was 11, while the number of predicted excitatory RNs was 14^(^^3^^)^. All but one of the pr-AMG RNs was predicted to be excitatory, with varying degrees of likelihood. This is different from our predictions in Reference (11), which indicated an inclination toward inhibitory pr-AMG RNs, but with low confidence. However, the predicted number of excitatory and inhibitory neurons in the RN group is closer to the observed number in Reference (3) than the predicted results from 3D point cloud matching, which suggests a promising direction for resolving the neurotransmitter types in that region. Further experiments and analysis are needed to determine with certainty the neurotransmitter type of each RN.

**Table 4.** Predicted neurotransmitter valence for the relay neurons (RNs). RNs used for training with known neurotransmitter type are omitted from this table and included in the Appendix. The column with our model predictions is “Network prediction,” which is “inconclusive” if there are fewer than three synapses for a given cell, or if the number of excitatory and inhibitory predictions are similar, that is, one is not more than 1.5 times the other.

Name	Cell group	Excitatory voxel	Inhibitory voxel	Excitatory score	Inhibitory score	Network prediction	Overlay estimation
90	Bipolar prIN	6,234	532	1.10E-02	1.40E-03	Excitatory	Excitatory
92	Bipolar prIN	6,767	259	1.20E-02	6.79E-04	Excitatory	Excitatory
93	Non-sensory RN	308	905	5.44E-04	2.37E-03	Inconclusive	Inconclusive
103	Non-sensory RN	5	867	8.84E-06	2.27E-03	Excitatory	Inconclusive
106	Non-sensory RN	51	1,924	9.01E-05	5.05E-03	Inconclusive	Inhibitory
122	Non-sensory RN	25	2,113	4.42E-05	5.54E-03	Excitatory	Inhibitory
125	Non-sensory RN	83	12,696	1.47E-04	3.33E-02	Inhibitory	Inhibitory
4	PNIN	0	0	0	0	Excitatory	Inconclusive
6	PNIN	0	0	0	0	Excitatory	Inconclusive
20	PNIN	0	0	0	0	Excitatory	Inconclusive
29	PNIN	0	0	0	0	Excitatory	Inconclusive
30	PNIN	0	0	0	0	Excitatory	Inconclusive
85	PNIN	6,909	484	1.22E-02	1.27E-03	Excitatory	Excitatory
61	PNIN	1,673	0	2.96E-03	0	Excitatory	Excitatory
65	PNIN	8,273	146	1.46E-02	3.83E-04	Excitatory	Excitatory
88	PNIN	13,979	236	2.47E-02	6.19E-04	Excitatory	Excitatory
131	PNRN	1	6,828	1.77E-06	1.79E-02	Excitatory	Inhibitory
74	pr-AMG RN	1,665	0	2.94E-03	0	Excitatory	Excitatory
94	pr-AMG RN	8,900	9,602	1.57E-02	2.52E-02	Excitatory	Inconclusive
108	pr-AMG RN	5,903	1,558	1.04E-02	4.09E-03	Excitatory	Inconclusive
116	pr-AMG RN	7,398	423	1.31E-02	1.11E-03	Inconclusive	Excitatory
124	pr-AMG RN	6,215	6,184	1.10E-02	1.62E-02	Excitatory	Inconclusive
127	pr-AMG RN	871	6,454	1.54E-03	1.69E-02	Excitatory	Inhibitory
140	pr-AMG RN	1,485	7,463	2.62E-03	1.96E-02	Excitatory	Inconclusive
157	pr-AMG RN	941	3,979	1.66E-03	1.04E-02	Inconclusive	Inconclusive
123	pr-BTN RN	6,077	2,991	1.07E-02	7.84E-03	Inconclusive	Inconclusive
130	pr-BTN RN	312	84	5.51E-04	2.20E-04	Inhibitory	Inconclusive
105	pr-cor RN	2,953	760	5.22E-03	1.99E-03	Inconclusive	Excitatory
112	pr-cor RN	197	6,530	3.48E-04	1.71E-02	Inconclusive	Inhibitory
119	pr-cor RN	236	3,234	4.17E-04	8.48E-03	Inconclusive	Inhibitory
80	prRN	2,301	1,352	4.07E-03	3.55E-03	Inconclusive	Inconclusive
86	prRN	4,822	1,696	8.52E-03	4.45E-03	Inconclusive	Inconclusive
96	prRN	3,660	4,863	6.47E-03	1.28E-02	Excitatory	Inconclusive
100	prRN	174	11,273	3.08E-04	2.96E-02	Inconclusive	Inhibitory
121	prRN	751	7,371	1.33E-03	1.93E-02	Inconclusive	Inhibitory
126	prRN	1,096	4,588	1.94E-03	1.20E-02	Inconclusive	Inhibitory

**Figure 9.**
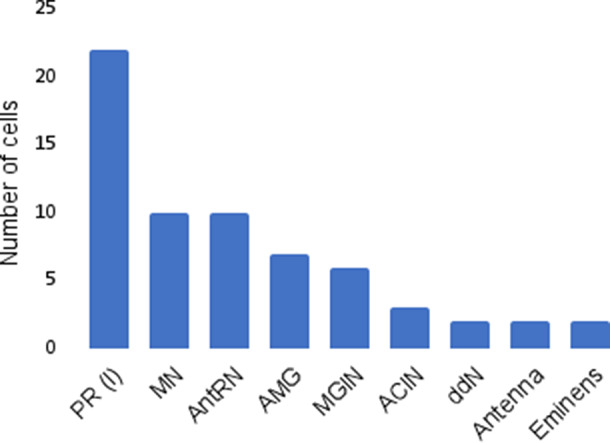
Number of unique cells per cell type of selected groups of interest.

**Figure 10.**
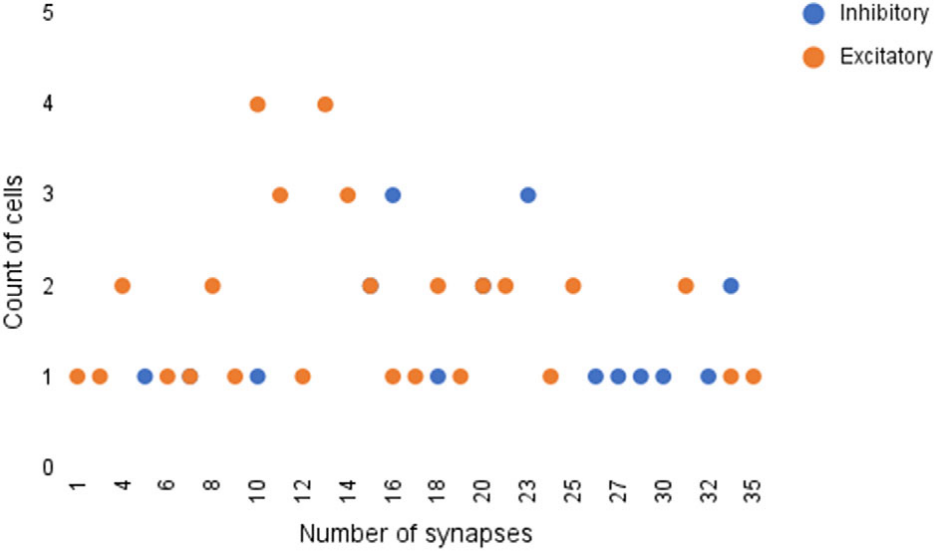
Frequency of cells with various number of synapses. It can be seen that the majority of cells have between 10 and 20 synapses.

The full prediction table is included in Table 5 in the Appendix. Summaries of the available data are shown in Figures 9 and 10.

### 5.1. Comparison with manual overlay

**Figure 11.**
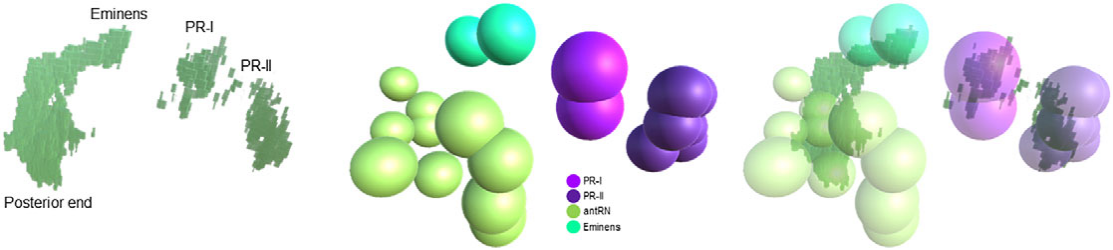
Illustration of manual overlay of in situ hybridization results for VGAT (left image) and EM-derived cell centroids (middle image) in 3D. Of the four cell groups shown, the areas with VGAT are encapsulated by the cell models, as seen in the rightmost image. See the text for more details.

**Figure 12.**
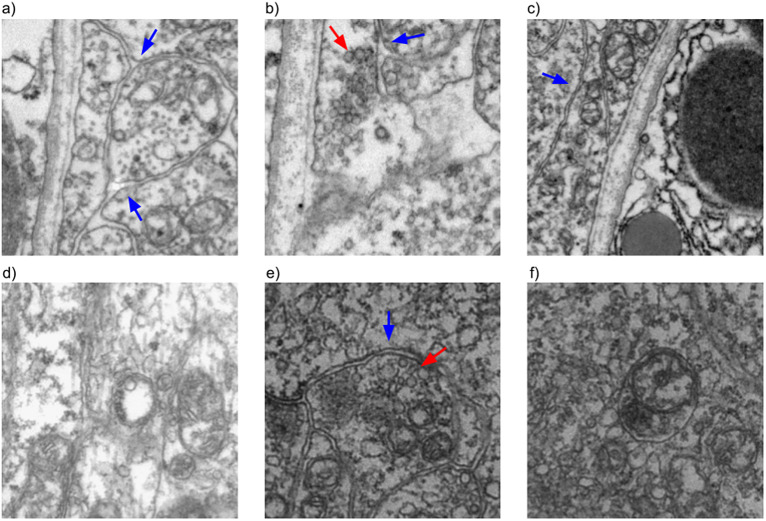
Examples of false positives for the synapse classification network. (a)–(c) contain coated vesicles, (d) and (e) contain botrysomes, and (f) contains an autophagosome. It can be seen that either cell boundaries (blue arrows) or groups of vesicles (red arrows) are visible in many of the cases.

The crux of our analysis lies in our hypothesis that the same cells appear in similar locations across *Ciona* specimens. To provide an additional point of comparison, we manually overlay the fluorescent imaging results from in situ hybridization with the cell centroids provided by the annotated EM data, as seen in Figure 11. Using the Unity software^(^^20^^)^, the centroid and volume of each neuron in the four selected cell groups as given in Reference (1) are rendered in 3D. Each neuron is approximated with a sphere of its corresponding volume. As seen on the leftmost image in Figure 9, image stacks of in situ from Reference (3) that contain expression of both vesicular GABA transporter (VGAT, inhibitory) and vesicular acetylcholine transporter (VAChT, excitatory) in the posterior Brain Vesicle are also rendered into the software with real-world dimensions. Since certain parts of the VGAT structure are well known and consistent, such as in the photoreceptors and at the posterior end of the RNs, this was used to manually align the in situ with the connectome. The matching criteria are as follows: the posterior border does not extend beyond the most posterior antRN, the dorsal cap marks the Eminen cells, and the two patches, a smaller posterior one and a larger anterior one, on the right mark the two photoreceptor groups, PR-I (only pr9 and pr10) and PR-II, respectively. After this alignment is done, the smaller VAChT-labeled regions are brought into view for analysis. Seven in situs from Reference (3) are aligned using the mentioned structures and similarity across the in situs as guides. Once they are aligned, a collision detector is used to compute the number of voxels in contact with each neuron. An illustration of the matching process is shown in Figure 11, and the comparative results are shown in Table 4.

## 6. Discussion

The proposed model has good performance on the classification of synapses in EM for *Ciona.* While there are some false positives from the model prediction (Table 1), this is desirable compared with false negatives, because an expert can then screen the predictions to determine true synapses. The ratio of synapses to nonsynaptic structures in a typical EM of *Ciona* is on the order of 1:1,000. Instead of scanning an entire image for possible synapses, the model can drastically reduce the annotation time needed for synapse annotation. The synapse prediction model has helped to identify possible neurotransmitter types for cells from certain neuronal groups, which were previously unknown. While we cannot be absolutely certain that the model has predicted correctly for synapses belonging to cells with previously unknown neurotransmitter types, the output of the model seems reasonable given the feature analysis we have done. Comparison with previous in situ hybridization results have also shown that the prediction of the model is likely correct. For the RNs, in Reference (3), we had previously found an average of 16 VGAT-positive neurons and 11 VAChT-positive neurons. The prediction of the network matches these numbers well, and much better than the point-cloud registration method used in Reference (3). We hope that the model predictions will work in conjunction with both prior and future analyses to help resolve the neurotransmitter type of individual neurons in regions that have been difficult to resolve using in situ hybridization and other experimental methods.

More work can be done on the analysis of cellular and subcellular features in neurons with undetermined neurotransmitter type. Overall connectivity and cell shape can be obtained from the annotated EM data and may be useful tools in better understanding the relationship between structural features and neurotransmitter-type expression. The connection between cell group and synapse structure can also be further explored, and may help with a more robust neurotransmitter prediction method. Additionally, detailed cell segmentation annotations could be created and added as another potential feature for analysis.

## Data Availability

Data and materials are available upon request.

## Appendix

**Table 5:** Ground truth predictions.

Cell ID	In situ	LLR test	LLR train	Excitatory test	Inhibitory test	Excitatory train	Inhibitory train	Prediction test	Prediction train
ACIN1L	Gly	0.24	−1.73	1	0	0	6	Excitatory	Inhibitory
ACIN2L	Gly	−0.68	0.12	1	2	4	3	Inhibitory	Excitatory
ACIN2R	Gly	−0.46	−0.54	0	1	1	3	Inhibitory	Inhibitory
AMG1	GABA	−1.08	−1.66	0	5	1	9	Inhibitory	Inhibitory
AMG2	GABA	−0.67	−3.09	1	4	1	14	Inhibitory	Inhibitory
AMG3	GABA	−0.21	−3.59	0	1	0	14	Inhibitory	Inhibitory
AMG4	GABA	−0.47	−2.42	0	2	2	12	Inhibitory	Inhibitory
AMG5	Ach	2.49	2.87	8	0	14	2	Excitatory	Excitatory
AMG6	GABA	−0.49	−3.55	1	2	1	14	Inhibitory	Inhibitory
AMG7	GABA	−0.6	−3.1	0	3	1	16	Inhibitory	Inhibitory
Ant1	Glut	0.21	3.89	3	1	14	0	Excitatory	Excitatory
Ant2	Glut	2.46	7.25	9	0	25	1	Excitatory	Excitatory
120	GABA	−1.56	−11.75	0	4	1	29	Inhibitory	Inhibitory
134	GABA	−1.58	−6.74	0	5	1	20	Inhibitory	Inhibitory
135	GABA	−1.15	−5.12	0	3	1	19	Inhibitory	Inhibitory
142	GABA	−1.54	−7.14	0	5	2	23	Inhibitory	Inhibitory
143	GABA	−1.75	−6.55	0	5	3	20	Inhibitory	Inhibitory
147	GABA	−2.94	−7.12	0	7	0	20	Inhibitory	Inhibitory
152	GABA	−2.58	−5.79	0	8	3	21	Inhibitory	Inhibitory
153	GABA	−1.52	−3.36	0	5	1	10	Inhibitory	Inhibitory
159	GABA	−1.79	−5.6	0	5	2	16	Inhibitory	Inhibitory
161	GABA	−3.02	−5.22	0	6	1	16	Inhibitory	Inhibitory
ddNL	Ach	1.39	4.69	4	0	16	0	Excitatory	Excitatory
ddNR	Ach	1.37	3.79	4	0	12	0	Excitatory	Excitatory
Em1	GABA	−1.11	−7.92	1	6	3	31	Inhibitory	Inhibitory
Em2	GABA	−1.6	−6.26	1	6	3	24	Inhibitory	Inhibitory
MGIN1L	Ach	0.87	6.27	5	0	25	1	Excitatory	Excitatory
MGIN1R	Ach	0.43	6	3	0	22	0	Excitatory	Excitatory
MGIN2L	Ach	0.82	4.85	3	0	14	0	Excitatory	Excitatory
MGIN2R	Ach	2.01	3.88	6	0	14	1	Excitatory	Excitatory
MGIN3L	Ach	0.33	2.91	1	0	7	0	Excitatory	Excitatory
MGIN3R	Ach	0.68	2	2	0	7	1	Excitatory	Excitatory
MN1L	Ach	1.13	5.77	3	0	18	0	Excitatory	Excitatory
MN1R	Ach	2.9	3.85	8	0	16	1	Excitatory	Excitatory
MN2L	Ach	1.84	3.46	4	0	9	0	Excitatory	Excitatory
MN2R	Ach	1.56	4.81	4	0	11	0	Excitatory	Excitatory
MN3L	Ach	0.43	1.91	1	0	5	0	Excitatory	Excitatory
MN3R	Ach	0.85	2.77	3	0	7	0	Excitatory	Excitatory
MN4L	Ach	1.91	1.02	6	0	4	0	Excitatory	Excitatory
MN4R	Ach	0.76	2.92	2	0	7	0	Excitatory	Excitatory
MN5L	Ach	0.4	1.34	1	0	3	0	Excitatory	Excitatory
MN5R	Ach	0.37	2.54	1	0	6	0	Excitatory	Excitatory
pr1	Glut	1.5	2.05	4	0	9	0	Excitatory	Excitatory
pr11	Glut	0.67	2.41	3	0	10	0	Excitatory	Excitatory
pr12	Glut	0.85	4.72	3	0	16	0	Excitatory	Excitatory
pr13	Glut	1.1	2.96	3	0	11	0	Excitatory	Excitatory
pr14	Glut	0.16	–	1	0	0	0	Excitatory	Excitatory
pr15	Glut	1.98	7.33	7	0	27	0	Excitatory	Excitatory
pr16	Glut	0.89	1.83	3	0	7	0	Excitatory	Excitatory
pr17	Glut	0.44	3.35	2	0	13	0	Excitatory	Excitatory
pr18	Glut	0.14	0.58	1	0	2	0	Excitatory	Excitatory
pr19	Glut	1.38	1.48	5	0	7	0	Excitatory	Excitatory
pr2	Glut	0.39	1.3	2	0	6	0	Excitatory	Excitatory
pr20	Glut	2.92	5.94	9	0	22	0	Excitatory	Excitatory
pr21	Glut	0.43	2.18	2	0	9	0	Excitatory	Excitatory
pr22	Glut	0.58	2.4	3	0	10	0	Excitatory	Excitatory
pr23	Glut	1.06	4.16	4	0	14	0	Excitatory	Excitatory
pr3	Glut	0.54	3.06	2	0	12	0	Excitatory	Excitatory
pr4	Glut	0.66	–	3	1	0	0	Excitatory	Excitatory
pr5	Glut	0.85	2.41	3	0	8	0	Excitatory	Excitatory
pr6	Glut	0.75	2.14	2	0	9	0	Excitatory	Excitatory
pr7	Glut	1.18	4.82	4	0	16	0	Excitatory	Excitatory
pr8	Glut	0.66	2.7	3	0	11	0	Excitatory	Excitatory
pr9	GABA	−0.36	−1.3	0	1	4	11	Inhibitory	Inhibitory
