# Automatic classification and neurotransmitter prediction of synapses in electron microscopy - CORRIGENDUM

**DOI:** 10.1017/S2633903X23000016

**Published:** 2023-02-01

**Authors:** Angela Zhang, S. Shailja, Cezar Borba, Yishen Miao, Michael Goebel, Raphael Ruschel, Kerrianne Ryan, William Smith, B. S. Manjunath

The authors would like to apologise for an error in the above article.

The Funding Statement originally omitted US National Science Foundation Award #1664172. This has been corrected.
